# Smart Robotics for Automation

**DOI:** 10.3390/s24123900

**Published:** 2024-06-16

**Authors:** Felipe N. Martins

**Affiliations:** Sensors and Smart Systems Group, Institute of Engineering, Hanze University of Applied Sciences, 9747 AS Groningen, The Netherlands; fe.nascimento.martins@pl.hanze.nl

In recent years, the demand for efficient automation across various sectors has accelerated significantly. E-commerce, for instance, has undergone rapid global expansion, particularly since the onset of the COVID-19 pandemic [1]. Smart robots offer potential solutions to cope with this surge in demand by enhancing warehouse efficiency. Moreover, robots can address various pressing issues stemming from diverse factors. The phenomenon of aging populations [2] signifies an increasing need for assistance among a larger demographic, while simultaneously leading to a decline in the available workforce. Aging populations present a spectrum of challenges, ranging from maintaining individual independence [3] to addressing the rising incidence of vision loss [4], which exerts significant strain on healthcare systems [5]. Climate change is already impacting food production [6,7] and is poised to exacerbate challenges leading to the displacement of entire populations [8], thereby precipitating a surge in demand for global infrastructure projects. These challenges underscore the critical need for innovative solutions in robotics, automation, and sensor systems.

In response to these pressing issues, the collection “Smart Robotics for Automation” in *Sensors* explores cutting-edge research and developments in the field, with a particular focus on related sensory aspects. The fifteen articles featured in this collection span a diverse array of applications, each contributing to the advancement of specific domains.

In the realm of industrial automation and robotics, several papers offer novel solutions for enhancing efficiency and safety in manufacturing and logistics. From humanoid motion control algorithms to collision detection methods for collaborative robots, these contributions address the key challenges faced by industries seeking to automate their processes. Focusing on robot motor control, Arciuolo et al. introduce a novel proportional–integral–derivative (PID) controller algorithm called PID++, which applies minor adjustments based on the real-time encoder position input to achieve a stable, precise, adaptive control system for linear motion control, regardless of load (contribution 1). This computationally lightweight approach boasts adaptability and precision, making it suitable for a wide array of applications, from industrial to biomedical settings. In turn, Li et al. argue that accurate real-time identification is essential for servo controller design, and propose an intelligent parameter identification method for robot servo controllers, enhancing dynamic performance and stability (contribution 4). Using advanced integration and optimization techniques, their approach enables accurate real-time identification of critical controller parameters.

Considering robot control, Czubenko et al. present a collision detection solution for collaborative robots (cobots) using a simple neural network architecture (contribution 6). The authors argue that a virtual sensor based on such a network can be used to detect various types of collisions of cobots or other mobile or stationary systems, improving the safety of human–machine interactions without the need for expensive equipment. Jung et al. present a hybrid imitation learning framework that combines behavior cloning and state cloning methods to enhance the robotic manipulation task learning efficiency (contribution 2). Their approach outperforms traditional methods, offering faster training times and improved performance in tasks such as pick-up, pick-and-place, and stacking tasks. Mandischer et al. introduce consumer-centered item detection methods for industrial environments, emphasizing usability and efficiency (contribution 7). They focus on unsupervised segmentation coupled with machine learning methods for classification, presenting the full pipeline from calibration and segmentation to item classification in the industrial context. Their approach, leveraging multicamera networks, offers reliable item detection and classification even at considerable ranges, improving efficiency in logistics and manufacturing. Regarding automation, Kilikevivcius et al. investigate vibrational transportation methods using dynamic dry friction control, unveiling novel capabilities for object transportation (contribution 8). Their research offers practical solutions for handling and transportation tasks in manufacturing and logistics, enhancing productivity and flexibility.

Other articles address problems related to mobile robots. Nascimento et al. tackle the challenge of safe path planning by proposing algorithms based on Probabilistic Foam that are able to find short paths or high-clearance paths (contribution 3). The authors present simulation studies to analyze the behavior, performance, and safety aspects of the proposed methods. Regarding robot navigation, Fagundes et al. propose an analytical formalism for object detection using 2D LiDAR sensors (contribution 15). Their research facilitates object detection and object localization in mobile robotics applications, offering a standardized approach for data representation using 2D LiDARs. Finally, robot localization is addressed by Klein et al., who present a machine learning approach to robot localization based on computer vision (contribution 14). They show the effectiveness of their solution in a competition scenario, paving the way for AI-driven localization in industrial environments.

Environmental sensing using unmanned aerial vehicles (UAVs) is addressed by two articles. Tanveer et al. explore the use of biosonar echoes for environment estimation in UAVs (contribution 5). Their simulation study demonstrates the effectiveness of biosonar-based techniques in accurately assessing foliage distributions, crucial for safe navigation in challenging outdoor environments. Zhou et al. address air quality monitoring challenges with fixed-wing UAVs, offering improved coverage and endurance compared to rotary vehicles (contribution 13). They present the development of a 3D coverage path planning method that enhances the spatial and temporal resolution for urban air quality profiling, aiding in environmental monitoring efforts.

In the domain of agriculture and aquaculture, some articles present robotic systems tailored for tasks such as vineyard surveillance and aquaculture inspection. Kapetanovic et al. present a heterogeneous autonomous robotic system tailored for viticulture and mariculture applications (contribution 11). In viticulture, they apply an all-terrain mobile manipulator and an autonomous aerial robot that can be used in very steep vineyards, where other mechanization fails. In mariculture, they also apply an autonomous surface vehicle and a remotely operated underwater vehicle. Their integrated approach can improve the efficiency of agricultural practices and reduce the amount of labor-intensive tasks such as vineyard surveillance, spraying, and aquaculture net pen monitoring. For aquaculture, Akram et al. present the integration of remotely operated vehicles with visual servoing for inspection of net pens (contribution 12). They propose a vision-based positioning system composed of an object detector, a pose generator, and a closed-loop controller. Their system offers a low-cost and efficient solution for monitoring underwater structures, ensuring the health and stability of fish farms.

Two articles highlight the capabilities of humanoid robots in sporting activities and human–robot interaction scenarios. Park et al. showcase the prowess of humanoid robots in sporting activities, particularly in skiing competitions (contribution 9). Their work highlights advancements in vision recognition and control, enabling humanoid robots to navigate challenging terrains autonomously. Gong et al. present a human–robot interaction method for real-time motion imitation focusing on a Tri-Co Robot (coexisting–cooperative–cognitive robot), facilitating natural and intuitive robot control (contribution 10). They use a motion capture system, which captures human motion and links it with a simplified skeleton model. Their dynamic time warping model enables precise motion evaluation, enhancing robot adaptability in diverse environments.

In conclusion, the papers featured in this collection represent a diverse array of innovative approaches and solutions to challenges in robotics and automation. The articles show that we can address pressing societal issues by harnessing the power of smart robotics and sensor technologies. We extend our gratitude to the authors for their contributions and hope that this collection will inspire further advancements in the field of sensors for robotics and automation.
